# Perceptions of Pre-exposure Prophylaxis (PrEP) for HIV prevention among men living with HIV in the context of reproductive goals in South Africa: a qualitative study

**DOI:** 10.1186/s12889-024-18118-4

**Published:** 2024-02-22

**Authors:** Xolani Ntinga, Oluwaseyi O. Isehunwa, Lindani I. Msimango, Patricia M. Smith, Lynn T. Matthews, Alastair Van Heerden

**Affiliations:** 1 Centre for Community Based Research, Human Sciences Research Council, Old Bus Depot, Sweetwaters, Pietermaritzburg, 3201 South Africa; 2 Division of Infectious Diseases, Department of Medicine, University of Alabama at Birmingham, Birmingham, AL USA; 3 SAMRC/WITS Developmental Pathways for Health Research Unit, Department of Pediatrics, School of Clinical Medicine, Faculty of Health Sciences, University of the Witwatersrand, Johannesburg, Gauteng, South Africa

**Keywords:** HIV prevention, Men living with HIV (MWH), Pre-exposure prophylaxis (PrEP), Reproductive goals, Reproductive health, Serodifferent couples

## Abstract

**Background:**

Pre-exposure Prophylaxis (PrEP) and Treatment as Prevention (TasP) are effective strategies to prevent HIV transmission within serodifferent couples. However, limited usage of PrEP, knowledge and interest has been amongst the barriers for men, alongside testing and treatment adherence. We explored the perceptions of PreP for HIV prevention with Men living with HIV (MWH) who have reproductive goals, to understand awareness and experiences related to PrEP use in the context of HIV prevention with their partners.

**Methods:**

We undertook a qualitative study with 25 MWH aged 18 to 65 between April and September 2021 in South Africa. Potential participants were screened for eligibility and scheduled to participate in telephonic interviews. Interviews were audio recorded, transcribed, translated and thematically analysed.

**Results:**

Themes were organized into opportunities and barriers that men with HIV articulate as important for using PrEP to meet individual, couple, and community reproductive goals. At the individual level, some men were willing to discuss PrEP with their partners to protect their partners and babies from acquiring HIV. Lack of knowledge about PrEP among men was a potential barrier to promoting PrEP among their female partners. At the couple level, PrEP use was seen as a way to strengthen relationships between partners, signifying care, trust, and protection and was seen as a tool to help serodifferent couples meet their reproductive goals safely. At the community level, PrEP was viewed as a tool to promote HIV testing and prevention efforts, especially among men, but participants emphasized the need for more education and awareness.

**Conclusion:**

Despite PrEP implementation in South Africa, awareness of PrEP among men with HIV in rural areas remains low. Engaging MWH to support their partners in accessing PrEP could be an innovative strategy to promote HIV prevention. Additionally, providing men with comprehensive reproductive health information can empower them to make more informed decisions, adopt safer sexual practices, and challenge societal norms and stigmas around HIV.

## Introduction

South Africa’s 8.45 million people with HIV (PWH) constitute the largest population of PWH in the world, with a national HIV prevalence rate of 14% in 2022 [1]. While HIV incidence is predominantly among women of reproductive age in Sub-Saharan Africa (SSA) [2, 3], HIV-serodifferent couples, where one partner is living with HIV and the other is not, play an important role in the epidemic. These couples face an increased risk of HIV transmission, accounting for up to half of up to half of all new HIV infections in Africa [4, 5]. Prevention efforts such as Pre-Exposure Prophylaxis (PrEP) and Treatment as Prevention (TasP), can prevent sexual transmission within HIV-serodifferent couples [6–8]. However, barriers to testing, treatment, and care for men who have sex with women [8, 9] have contributed to limited use of these prevention methods, particularly PrEP [7]. Engaging men and their partners in reproductive health can promote behavioural changes that reduce the risk of HIV transmission, such as increased condom use, HIV testing, and status disclosure to partners [10]. Additionally, addressing structural drivers of HIV among women, such as poverty and gender-based violence, can help create an enabling environment for women to make informed reproductive health choices and reduce their risk of HIV [11].

Reproductive goals, such as family planning, can be a powerful opportunity to engage men and partners to prevent HIV among women of reproductive age [12]. In previous work, members of this team developed and piloted a safer conception intervention *“Sinikithemba Kwabesilisa*” meaning *we give hope to men*, designed for men with HIV planning to have children with an HIV uninfected partner [10, 13]. We showed feasibility, acceptability, and preliminary data that the intervention successfully supported men with HIV (MWH) to access ART and suppress viral load [14, 15]. However, MWH were recruited from an urban setting and PrEP use was not explored. Considering these prevention opportunities, we conducted a qualitative study focused on reproductive goals to engage men and their partners and explored awareness and experiences related to PrEP. By investigating the potential opportunities for the implementation of PrEP and other HIV prevention strategies within the context of reproductive goals, we sought to better understand the unique challenges they face, the potential influence they have on preventing HIV transmission to their partners, and how best to integrate prevention strategies that align with their reproductive goals.

### Study design and study population

We conducted an independent qualitative study involving in-depth telephonic interviews (IDIs) with men with MWH in KwaZulu-Natal, South Africa. KwaZulu-Natal has the highest provincial HIV prevalence, and uMgungundlovu District is among the districts with the highest prevalence in the country at 30% [16]. This study was conducted in the Greater Edendale area, uMgungundlovu District, KwaZulu-Natal, which has informal, rural, and peri-urban dwellings; the population is mainly isiZulu first language speakers.

## Methods

### Study participants

The Human Sciences Research Council maintains a database of people who have participated in HIV prevention and treatment research studies and provided written consent to be contacted for future research activities. Men living with HIV were recruited between April and September 2021 from the Human Sciences Research Council database. The community outreach team contacted these MWH aged 18 to 65, willing to be screened for potential participation in the study and fluent in either isiZulu or English. Participants were eligible to participate in the study if they met the eligibility criteria - identified as heterosexual male, aged between 18 and 65 years, living with HIV and in uMgungundlovu District, and fluent in either English or isiZulu. A total of 50 men in the database were randomly contacted for screening, 30 of the 50 men screened met eligibility criteria. Of these 30 men, 4 could not be reached again and 1 declined a telephonic interview (preferred a face-to-face interview). The final 25 men who participated in this study were already engaged in care and most had been on treatment for at least 2 years.

### Procedures

People willing to be screened were contacted by one of the co-authors, LM, a Black South African first language isiZulu speaker and a research assistant with qualitative methods training, who also received training on the study interview guide. LM explained the purpose of the study and answered participant’s questions about the study. This study was conducted at the height of the COVID-19 pandemic, and to mitigate transmission risks, data procedures were conducted telephonically. During the first phone call, the researcher reviewed the virtual data collection procedures including the informed consent process, and obtained verbal consent. A physical copy of the informed consent form was delivered to enrolled participants on the same day of consenting by the community outreach team and the interview was scheduled for between 1 and 3 days after the consent was delivered. Each participant was reimbursed with ZAR150 (~ 11 USD at that time) according to the time, inconvenience, and expenses model [16] that the National Health Research Ethics Committee endorses.

### Data collection

Prior to the interview, a research assistant administered a brief demographic questionnaire that collected socio-demographic characteristics, HIV status, and reproductive history. The IDI guide comprised of questions to assess (1) perceptions around current HIV testing uptake and HIV disclosure, (2) perceptions around fatherhood, reproductive goals, and decision-making process, (3) attitudes, perceptions regarding antiretroviral treatment and living with HIV, (4) perceptions around, safer conception care including TaSP and PrEP, and (5) barriers to care and reproductive goals due to COVID-19 pandemic. IDIs were conducted telephonically in isiZulu and lasted 46–144 min.

### Data analysis

All interviews were audio-recorded, transcribed, and then translated into English. Data were analyzed based on the thematic analysis approach using both deductive and inductive codes. The research team read and discussed a few of the transcripts in detail to identify themes. Preliminary themes were reviewed and refined, and final themes were organized into a codebook comprising major and sub-themes, and descriptions. Using the codebook developed, two research team members (OI and XN) double-coded 10% of the transcripts to ensure accurate interpretation of the codes. The remaining transcripts were independently coded by OI and XN. NVivo 12 software was used to facilitate the organization, coding, and management of data.

## Results

Between April and September 2021, 30 men were screened for semi-structured interviews; 5 declined participation, with one citing their preference to do an in-person interview which was not possible considering the required COVID-19 precautions. Four potential participants did not respond when contacted for interviews. In total, 25 men were interviewed. Their median age was 44 (IQR 13), 16 (60%) were unemployed, 13 (52%) had completed high school. Fourteen (56%) had known their HIV-serostatus for at least 10 years, and all were accessing ART. Eighteen men (72%) reported a partner with HIV, and 7 (28%) reported an uninfected partner. Most (*N* = 22, 88%) reported having only one sexual partner, and 16 (64%) reported that they were in a long-term relationship with their partner (primary partner for at least one year or living together). —Table 1. The majority of the participants reported they had not previously heard about PrEP. The few participants who had heard about PrEP described hearing about it primarily from healthcare providers during their clinic visits. Other sources included the radio and public health facilities.

**Table 1:** Demographic characteristics of participants (*N* = 25)

Characteristics		Overall
Age	Median (min, max)	44 (28, 58)
Education	Completed primary school or below	2 (8%)
	Some secondary school	10 (40%)
	Completed secondary school or higher	13 (52%)
Employment status	Not employed	15 (60%)
	Part-time/Self-employed	10 (40%)
Race	Black South African	24 (96%)
	Black Non-South African	1 (4%)
Total number of partners past 6 months	1	22 (92%)
	2	1 (4%)
	3	1 (4%)
Duration of HIV diagnosis	< 2 years	4 (16%)
	2–5 years	3 (12%)
	5–10 years	4 (16%)
	> 10 years	14 (56%)
Accessing ART	Yes	25 (100%)
Number of children fathered	0	5 (20%)
	1	4 (16%)
	2	7 (28%)
	3+	9 (36%)
Relationship status with primary partner	Spouse/living as married	5 (20%)
	Long-term partner	16 (64%)
	Partner < 1 year	4 (16%)
Number of children with primary partner	0	15 (60%)
	1	3 (12%)
	2	5 (20%)
	3+	2 (8%)
Condom use with primary partner during last sex	Yes	13 (52%)
	No	12 (48%)
Do you want to have a baby with your primary partner?	Yes	17 (68%)
	No	5 (20%)
	Don’t know	3 (12%)
Do you think your main partner wants to have a baby with you?	Definitely yes (same combos suggested as above)	13 (52%)
	Probably yes	4 (16%)
	Probably not	2 (8%)
	Definitely not	3 (12%)
	Never discussed	3 (12%)
HIV status of primary partner	HIV positive	18 (72%)
	HIV negative	7 (28%)

Emerging themes are organized into opportunities and barriers that men with HIV articulate as important for using PrEP to meet reproductive goals at the individual, couple, and community level. The analysis was structured to examine the knowledge and use of PrEP among MWH and the importance of PrEP in supporting reproductive needs.

## 1. Individual level opportunities and barriers

### a. Men may be willing to discuss PrEP with their partner to protect their partner and baby from acquiring HIV

For the men who had heard about PrEP prior to this study, they expressed their desire to discuss PrEP use with their partner.

*Let me say this intervention is already implemented and I have seen it, we have it at the clinic. Many people who utilised it did not have any complains about it. I did discuss this with my partner, and I could see that she was not interested, so I ended up giving up on it; but I know this intervention is very effective. If it was me who was not infected and she was, I would take the treatment” (M003).*

*They (clinic staff) introduced it the way that you did. They mentioned that it was aimed at reducing the spread of HIV and that those without HIV can use this treatment to prevent being infected if they have partners who are infected with HIV. What I don’t know is whether many people utilize it, but overall, it was a good intervention. (M003)*

### b. Lack of knowledge about PrEP among men living with HIV may be a barrier to promoting PrEP among their female partners to fulfil their reproductive goals

Many participants in the study revealed a lack of prior knowledge about PrEP which was perceived as a potential barrier for MWH failing to meet their reproductive goals. One participant highlighted; *“Some people die without having children because they don’t have this information.”* (M011).

They emphasized that enhanced education and awareness will ensure that men are aware of PrEP and its effectiveness in preventing HIV transmission.

*“It could really work for them, but the problem is that the majority of men don’t know or are not aware about this pill that you are talking about. And even though there are some men who know about it, but I think their knowledge is very limited… I wish that health workers would conduct health awareness where people would be educated about this, because it is very important.” (M006)*.

Another participant added, *“People only know about ARVs they don’t know that there is a pill you can take to prevent being infected with HIV” (M018).*

Some participants did possess knowledge about Prevention of Mother-to-Child Transmission of HIV (PMTCT) programme and the possibility of giving birth to an HIV negative child when a parent is living with HIV; however, they remained less knowledgeable about other HIV prevention methods available to protect against HIV transmission between partners prior to pregnancy.

*“I know that you can have a child and the child does not get infected if you take medication. However, I am not sure how you would protect your partner” (M021).*

For those who had detailed knowledge about PrEP, they mentioned acquiring this information from various sources such as radio or during their clinic visits as well as from other healthcare professionals outside clinic facilities as one participant shared:

## 2. Couple level opportunities and barriers

### a. Opportunities to deepen relationships with their female partner may encourage men to support their partners to use PrEP

Participants described how PrEP use could help strengthen relationships signifying care, trust and protection between partners. One participant expressed this by saying:

*“I think that it would help and very few relationships would break. It will also allow people to build trust when all precautions have been taken. So, it is important that the couple protects themselves because at the end of the day, they want to have children. I think it will really help” (M021).*

Another participant emphasized the collaborative aspect of PrEP use among couples:

*“I think that will be okay because both partners will be responsible for each other to take the pills. They might work together and support each other to take their respective treatment every day because if you love each other you always want to protect one another” (M019).*

### b. Opportunities to safely meet reproductive goals

Men felt that PrEP could help those in HIV-serodifferent relationships be more supportive towards each other in remaining healthy to achieve their reproductive goals.

*“I think it would and help very much because it also gives the other partner who is negative courage to consider having children. This will be helpful to protect the one who is not infected whilst also validating the other individual who is infected” (M027).*

Another participant highlighted the importance of allowing individuals with HIV to provide PrEP to their partners, saying:

*“That will depend on whether as an HIV positive person I am allowed to give that pill to my partner. Other people do not even want to hear about it. If I am allowed to have the pill and I will tell that person that since we are not going to use a condom please take this pill, then I can give it to my partner. People who have HIV should be given this pill to give to the person they are with at that time, but this must not encourage us to be promiscuous.” (M007)*.

Furthermore, they believed PrEP could decrease viral load and strengthen the immune system, providing people living with HIV the confidence to have children without the fear of transmitting to their partners:

*“Yes, it will be effective; it can prevent transmission, it means the viral load will be decreased and the immune system will be stronger. And people living with HIV will not infect people intentionally. It will also help men living with HIV to still have children, and not fear infecting their partner with HIV. Hence, it can give people courage.” (M025).*

One participant described how PrEP use could benefit couples in serodifferent relationships with a desire to have children.

*“It would depend on the couple, if they want to have children then it would work for them” (M017).*

Further to PrEP being beneficial to couples in serodifferent relationships; some participants perceived taking PrEP as one step towards ending the HIV epidemic and also making people aware that they could conceived healthy HIV negative children.

*“Everything would be okay. Things might even go back to a situation where the virus is something that we can get rid of. People would not be scared that they are going to give birth to children that are going to die” (M007).*

### b. Fear of rejection and HIV related stigma may hinder men living with HIV to discuss PrEP with their partners

Participants believed that PrEP could have negative or positive consequences among couples in HIV-serodifferent relationships.

*“It would depend on the intensity of their love, because if that’s not the case, if they have a disagreement the one who is not HIV positive might say mean things to the one who is HIV positive. So, it’s all about how dedicated they are to each other. The one on PrEP is supporting the one living with HIV and showing how committed he or she is. The one living with HIV can see that taking PrEP is a manifestation of love, otherwise the person would have left. Just like if a man living with HIV wants to have a child, he can tell his partner about PrEP” (M025).*

Another participant felt that PrEP would be met with skepticism from the un-infected partners, about the need to taking pills if they are HIV negative.

*“I think that women would not trust this pill, many might ask why they should take the pill if they are negative” (M017).*

## 3. Community level opportunities and barriers

### a. PrEP could be a tool to promote HIV testing and prevention efforts especially among men

One participant mentioned that even though PrEP would benefit the community, the issue of men not knowing their status is key, given that most men do not know their status, and for them to benefit from PrEP, more must be done to educate people, especially men.

*“Yes, I think this may be a viable option provided that men and women still get tested to know their status before just saying they are negative and starting on PrEP. Yes so, I really think if people were to really get informed a bit more about PrEP, this is the one option that I feel may be viable for them to use over the other one because as you are explaining, it seems like it’s something that could really work. However, it all depends on the person knowing their status because if you know your status then it makes it easier for you to know what kinds of preventative measures are available for you to use.” (M026)*.

Men expressed the desire for more education and awareness around HIV prevention methods and safer conception care in clinics and community spaces including community halls, schools.

*“It would help a lot; they can talk to people about this at the Clinic or at the community hall and educate them about this.”* (M007).

*“It should happen in the community; assistance must be brought to the community. Other people cannot afford taxi or bus fare to get to the health centers. Hence, they must come to a community”* (M022).

*“I think such discussions need to happen where most people meet. However, since COVID prevents people from meeting in masses, the clinic would be okay. This could happen while people are on the queue, they could be some someone educating people while they wait.… I think that’s the perfect place, because when people are called to awareness programmes they usually don’t come in large numbers. So, I see the clinics and hospitals as the best place for conducting awareness programmes since many people go there. Even if there are there is few people, those few people will go back home and educate others, thus many people will get the word.”* (M006).

*“I think you could put up posters at the hall and look for a hall that could accommodate everyone. I think people would appreciate it. People are sick and they want help. They would appreciate the information. Some people die without having children because they don’t have this information”* (M011).

## Discussion

In this present study, we sought to explore perceptions around PrEP including awareness and acceptability among men living with HIV in South Africa within the context of their reproductive goals. Although PrEP implementation and roll out in South Africa began in 2015 [17], and its effectiveness in preventing HIV transmission is well documented in previous studies [18–21] findings from this study suggests a low PrEP awareness among rural South African men with HIV where a third were in sero-different relationship and desire to have a child with their primary partner. Findings are consistent with other studies on PrEP among men and women living with HIV conducted in South Africa and other African countries [13, 22, 23] While these men in the study are all actively engaged in HIV care, there’s an expectation that they should be knowledgeable about PrEP as an HIV prevention, this suggests that HIV prevention implementers, including clinicians should explore the reproductive goals of men in the context of HIV as a tool to promote HIV prevention, as men who are motivated to have healthy babies are more likely to engage in honest conversation about their HIV status with their partners and allow for shared decision making about safer conception practices including TasP and PrEP and reproductive planning [13]. This study also suggests that community outreach is a key strategy that should be implemented to ensure that knowledge about Prep is accessible to people who may not typically visit clinics, which is where most people who need PreP are likely to be found. Community outreach allows for culturally tailored messaging and education to ensure the message resonates with the community members [24, 25].

Participants in our study also expressed mixed attitudes and support for PrEP after a detailed description of PrEP was given. While some participants expressed support for PrEP and perceived it could be beneficial for those in serodifferent relationships with plans to have a child, some others were skeptical about the need for an un-infected partner to use PrEP. In addition, some expressed concerns about the availability of PrEP, consistent use, and possible side effects of PrEP. In contrast, these findings are different from a local study conducted among men living with HIV in urban KwaZulu province [15, 26]. However, in the study, men living with HIV had completed a 12-week safer conception program that included safer conception counseling, education, communications, and problem-solving skills building province [15, 26].

While young women in Africa are more vulnerable and disproportionately affected by HIV [2, 3], men living with HIV could play a key role in ending HIV. Given the power structure in African communities, many men in heterosexual relationships still drive key decisions in relationships, especially around condom use and conception plans. However, challenges to engaging men in care remain. Therefore, providing men living with HIV with comprehensive reproductive health information, including safer conception strategies, HIV transmission, risk and the importance of medication adherence allows them to make more informed decisions and encourages them to adopt safer sexual practices that reduce the risk of HIV transmission and protect their partners [10]. When men are supported in their reproductive desires that helps challenge some societal norms and stigmas around HIV as these men engage in seek testing behaviours, HIV testing, adhere to treatment and improve their involvement in care [12, 15]. Tailoring reproductive health programs to cater to men’s needs can also help overcome barriers to testing, treatment, and preventive measures [16]. Having men around reproductive health discussions can also help shift gender dynamics and while promoting more equitable relationships, as men who participate in reproductive planning can be exposed to discussion about contraception, safer sex practices and family planning which ultimately promote HIV prevention [15].

### Strengths and limitations

By exploring men living with HIV (MWH) and their perspectives of Pre-Exposure Prophylaxis (PrEP) and their reproductive aspirations, the study addressed a significant gap in the literature and highlighted the critical relationship between PrEP awareness and reproductive health goals. This connection emphasized the role that reproductive health plays in getting males involved in HIV prevention measures and offered possible avenues for interventions and programs that aim to integrate reproductive health education into HIV prevention initiatives. However, there are several limitations of this study. First, only MWH from rural KwaZulu Natal, South Africa, were part of this qualitative study; men from other regions, men in urban settings, and the viewpoints of female partners were not included… Second, because of the COVID epidemic, interviews were done over the phone. Because the majority of the participants were from semi-rural locations, several of them experienced difficulties with network connections, or their batteries died during the interview. Furthermore, even though the interviews took place following multiple interactions with the participants, building rapport over the phone has significant limitations. Nonetheless, there was no variation in the overall quality of the data compared to our in-person interview experience. Finally, even though this study’s sample size was 25 MWH, we were nevertheless able to get rich insights from the participants about opportunities and challenges of engagement in HIV care.

### Recommendations for future research

Although the study provided insightful information, more research is necessary to fully explore or validate the other aspects or viewpoints that may influence MWH’s awareness of and use of PrEP. This can be done by larger scale studies or complementing quantitative research.

## Conclusion

In this population of research-experienced, adult MWH, many diagnosed for more than a decade, PrEP knowledge was low in 2021, five years after the start of South Africa’s PrEP program. Men were optimistic about PrEP as an important prevention strategy for their partners and others in their community. Engaging MWH to support their partners to access PrEP may be a novel implementation strategy in the context of reproductive goals.

## Data Availability

The data sets generated and analyzed during this study are available from the corresponding author upon reasonable request.
